# Phosphorylation of Cysteine String Protein Triggers a Major Conformational Switch

**DOI:** 10.1016/j.str.2016.06.009

**Published:** 2016-08-02

**Authors:** Pryank Patel, Gerald R. Prescott, Robert D. Burgoyne, Lu-Yun Lian, Alan Morgan

**Affiliations:** 1 Department of Cellular and Molecular Physiology, Institute of Translational Medicine, University of Liverpool, Crown Street, Liverpool L69 3BX, UK; 2 NMR Centre for Structural Biology, Institute of Integrative Biology, University of Liverpool, Crown Street, Liverpool L69 3BX, UK; 3 Department of Biological and Environmental Sciences, University of Hertfordshire, Hatfield AL10 9AB, UK

**Keywords:** adult onset neuronal lipofuscinosis, chaperone, DnaJ, Hsp40, neurodegeneration

## Abstract

Cysteine string protein (CSP) is a member of the DnaJ/Hsp40 chaperone family that localizes to neuronal synaptic vesicles. Impaired CSP function leads to neurodegeneration in humans and model organisms as a result of misfolding of client proteins involved in neurotransmission. Mammalian CSP is phosphorylated in vivo on Ser10, and this modulates its protein interactions and effects on neurotransmitter release. However, there are no data on the structural consequences of CSP phosphorylation to explain these functional effects. We show that Ser10 phosphorylation causes an order-to-disorder transition that disrupts CSP's extreme N-terminal α helix. This triggers the concomitant formation of a hairpin loop stabilized by ionic interactions between phosphoSer10 and the highly conserved J-domain residue, Lys58. These phosphorylation-induced effects result in significant changes to CSP conformation and surface charge distribution. The phospho-switch revealed here provides structural insight into how Ser10 phosphorylation modulates CSP function and also has potential implications for other DnaJ phosphoproteins.

## Highlights

• First structure of a phosphorylated DnaJ/Hsp40 protein
• Phosphorylation destabilizes CSP's N-terminal α helix
• Newly disordered, phosphorylated N-terminal loop binds to the J domain
• Phosphorylation causes significant changes to CSP conformation and surface charge

## Graphical Abstract

**Figure:**
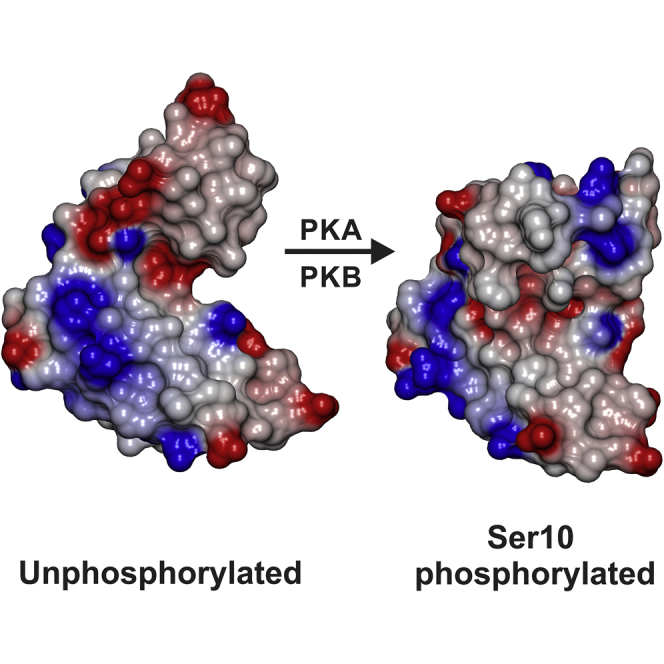

## Teaser

Cysteine string protein (CSP) is phosphorylated in vivo on Ser10, and this modulates its protein interactions and effects on neurotransmitter release. Patel et al. report that Ser10 phosphorylation disrupts CSP's extreme N-terminal α helix, which triggers formation of a hairpin loop stabilized by ionic interactions between phosphoSer10 and the J-domain residue, Lys58.

## Introduction

CSP is a member of the DnaJ/Hsp40 family of molecular chaperone proteins. It is highly expressed in all neurons, where it localizes to synaptic vesicle membranes (Chamberlain and Burgoyne, 2000). Mammals express three CSP isoforms (α, β, γ), but CSPα is the major brain isoform and is the ortholog of the single CSP expressed in invertebrates. Human CSPα is encoded by the *DNAJC5* gene, mutations in which cause the neurodegenerative disorder, adult-onset dominant neuronal ceroid lipofuscinosis (Noskova et al., 2011). As mutations in CSP-encoding genes also cause neurodegeneration in flies (Zinsmaier et al., 1994), worms (Kashyap et al., 2014), and mice (Fernandez-Chacon et al., 2004), it is clear that CSP performs a universal neuroprotective function (Burgoyne and Morgan, 2015). CSP is widely thought to prevent neurodegeneration by promoting the correct conformation of presynaptic proteins involved in synaptic exo/endocytosis. Compelling evidence suggests that the SNARE protein SNAP-25 is one such protein, whose misfolding in the absence of CSP leads to neurodegeneration (Burgoyne and Morgan, 2011, Sharma et al., 2011, Sharma et al., 2012). However, numerous other CSP-binding proteins have been suggested as functionally relevant client proteins for refolding, including the SNARE protein syntaxin (Chamberlain et al., 2001, Evans et al., 2001, Nie et al., 1999, Swayne et al., 2006); the calcium sensor synaptotagmin (Boal et al., 2011, Evans and Morgan, 2002); G protein subunits (Magga et al., 2000); and the endocytic protein dynamin (Zhang et al., 2012).

**Figure 1:**
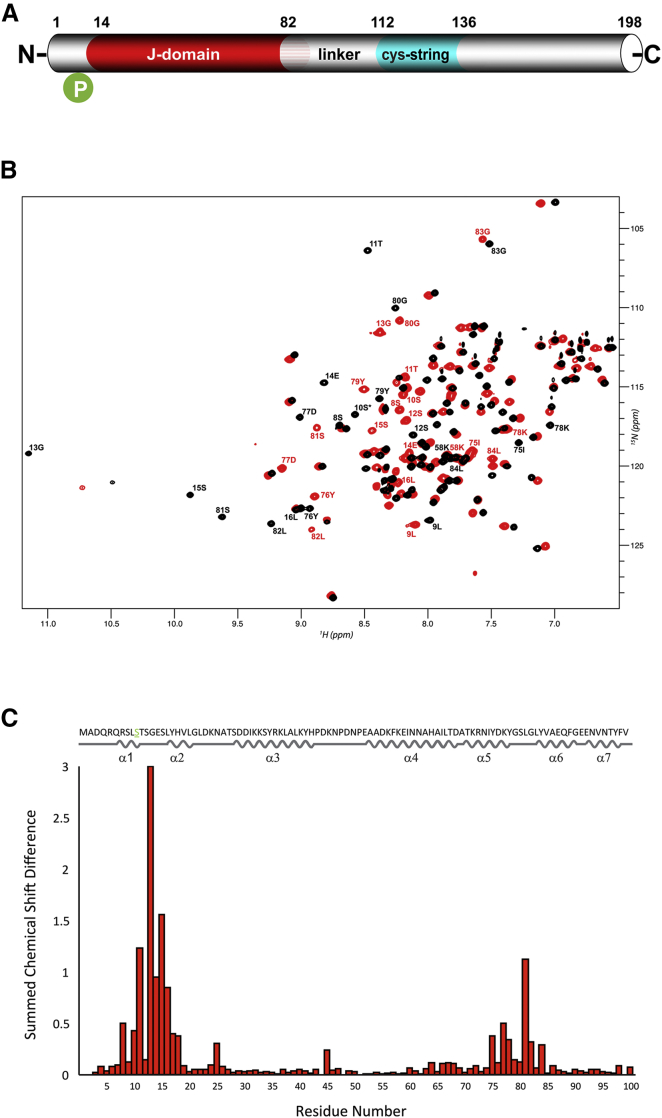
NMR Analysis of Unphosphorylated and Phosphorylated CSP_1-100_ (A) Domain structure of CSP. (B) ^1^H-^15^N HSQC spectra of CSP_1-100_ (red) and pCSP_1-100_ (black). The HSQC spectra shows well-resolved, non-overlapping peaks indicating both CSP_1-100_ and pCSP_1-100_ are folded. Upon phosphorylation, chemical shift dispersion can be observed for the indicated residues around Ser10 and Ser81. (C) Chemical shift differences (Δδ) between CSP_1-100_ and pCSP_1-100_ amide resonances, calculated using Δδ = [(δ_H_)^2^ + (δ_HN_^∗^0.15)^2^]^1/2^.The amino acid sequence and secondary structure elements of CSP_1-100_ obtained from the NMR structure are shown at the top of the figure.

CSP has an evolutionarily conserved domain structure (Figure 1A). The J domain is a signature of the DnaJ/Hsp40 family of molecular chaperones, which bind misfolded proteins and recruit/activate the 70 kDa heat shock cognate protein (Hsc70/Hsp70) to regulate protein folding (Hennessy et al., 2005). Indeed, CSP binds Hsc70 and stimulates its ATPase activity, and prevents aggregation of denatured proteins (Braun et al., 1996, Chamberlain and Burgoyne, 1997a, Chamberlain and Burgoyne, 1997b). All other domains are unique to CSP homologs. The cysteine string domain comprises 13–15 cysteine residues in an approximately 25-amino-acid motif, most of which are palmitoylated (Gundersen et al., 1994). This domain is essential for targeting CSP to synaptic vesicles and for neurotransmitter release in vivo (Arnold et al., 2004, Chamberlain and Burgoyne, 1998, Greaves and Chamberlain, 2006, Ohyama et al., 2007, Stowers and Isacoff, 2007). The function of the linker region connecting the J domain to the cysteine string is unclear, as mutation of this domain has relatively mild effects on CSP phenotypes (Arnold et al., 2004, Bronk et al., 2005, Zhang et al., 1999), although it may regulate binding to synaptotagmin (Boal et al., 2011). The C-terminal domain displays relatively low sequence conservation among CSP homologs from various species; and its function is poorly understood. Finally, CSPs contain a short N-terminal polypeptide sequence that is phosphorylated in vivo from worms to humans (Collins et al., 2005, Evans and Morgan, 2005, Evans et al., 2001, Hilger et al., 2009, Zielinska et al., 2009). Phosphorylation of mammalian CSPα on Ser10 inhibits binding to syntaxin and synaptotagmin, but not Hsc70, (Evans and Morgan, 2002, Evans et al., 2001) and modulates cellular exocytosis release kinetics (Chiang et al., 2014, Evans et al., 2001). However, there are no data on how Ser10 phosphorylation affects CSP structure to bring about these functional changes. Here we report the nuclear magnetic resonance (NMR) structures of the CSP N terminus in both the unphosphorylated and phosphorylated states.

## Results

### Generation of Soluble, Monomeric CSP Constructs for NMR

To investigate the structural consequences of phosphorylation on mammalian CSPα, we purified bacterially expressed recombinant proteins for analysis. Full-length CSP_1-198_ formed mixed oligomers of >239 kDa, based on analytical ultracentrifugation (AUC) analysis (Figure S1A), representing at least ten subunits based on the predicted monomeric mass of 23.5 kDa. In contrast, the C-terminal domain construct CSP_137-198_ was monodisperse with an estimated molecular mass of 9.0 kDa, close to its predicted monomeric mass of 8.2 kDa (Figure S1B). The heteronuclear single quantum coherence (HSQC) spectrum for ^15^N-labeled CSP_137-198_ shows poor ^1^H chemical shift dispersion, with most resonances appearing between 7.9 and 8.6 ppm, indicating that the C-terminal domain is essentially unstructured (Figure S1C). It has been suggested that CSP's tendency to aggregate may be due to the cysteine string domain (Swayne et al., 2003). However, mutation of all 14 cysteines to serines in full-length CSP_1-198_ did not reduce oligomerization (Figure S1D), and a CSP_1-112_ construct that lacks the entire cysteine string precipitated into visible aggregates. In contrast, CSP_1-100_ was monomeric with well-dispersed resonances in the ^1^H-^15^N HSQC spectra (Figure 1B). Further structural work was therefore performed using CSP_1-100_.

### Solution Structure of CSP_1-100_ in the Non-phosphorylated State

**Figure 2:**
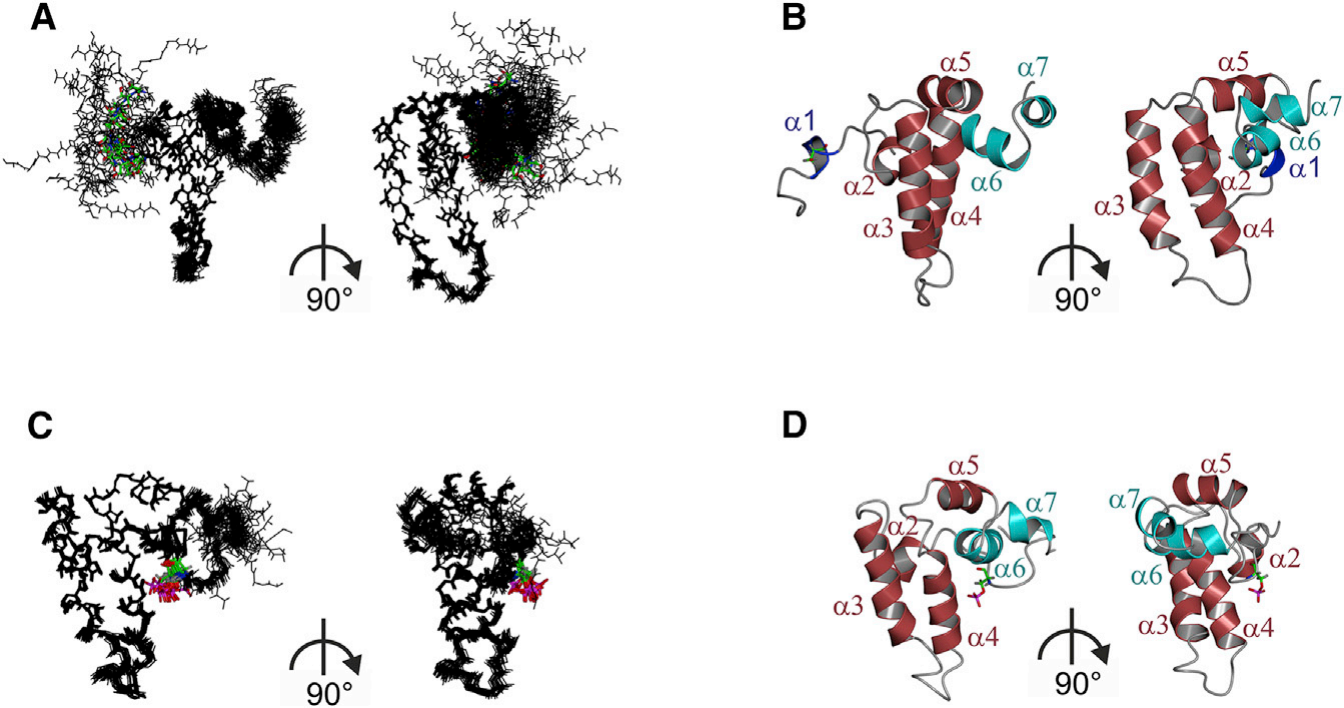
Structures of Unphosphorylated and Phosphorylated CSP_1-100_ Ensembles (A and C) and ribbon representations (B and D) of the lowest-energy conformers of CSP_1-100_ (A and B) and pCSP_1-100_ (C and D); two different views differing by 90° are shown for each, with Ser10 represented as sticks. For the ensembles, all main-chain heavy atoms for 20 structures are displayed (see also Figure S2 for Cα backbone ensembles). For the ribbon representations, helices are highlighted in purple (α1), red (α2–α5), or cyan (α6–α7).

Using conventional triple-resonance NMR spectra, the backbone resonances for 99 of 100 residues of CSP_1-100_ were assigned, and the structure was determined with 2,637 distance and dihedral angle restraints (Table 1). This revealed a secondary structure consisting of seven α helices, α1(7–10), α2(16–20), α3(28–42), α4(52–68), α5(70–78), α6(83–90), and α7(93–98) (Figure 1C). These secondary structure elements are well defined, although helices α1, α6, and α7 are much less converged than helices α2–α5 due to the lack of stabilizing helix-helix interactions in the tertiary structure (Figures 2A, 2B, and S2A). Helix α1 is a short α helix located in an otherwise highly flexible, unstructured N-terminal region; helices α2–α5 comprise the autonomously folded J domain; and helices α6 and α7 are located in the linker region C-terminal to the J domain. The secondary structure and overall fold of helices α2–α5 strongly resemble other previously determined J-domain structures, such as yeast Sis1p (PDB: 4RWU; Figure S3A). Our CSP_1-100_ structure is also similar to the structure deposited by the RIKEN Structural Genomics Consortium of a CSP_5-100_ construct (PDB: 2CTW; Figure S3B), although clear differences are apparent in the non-J-domain helices: α1, α6, and α7. This is especially evident in the N-terminal α1 helix, which is not helical in any of the 20 submitted 2CTW structures. It is likely that the first four residues of CSP, which are absent in the 2CTW construct, are important for α1 helix formation.

### A Phosphorylation-Induced Conformational Switch

**Figure 3:**
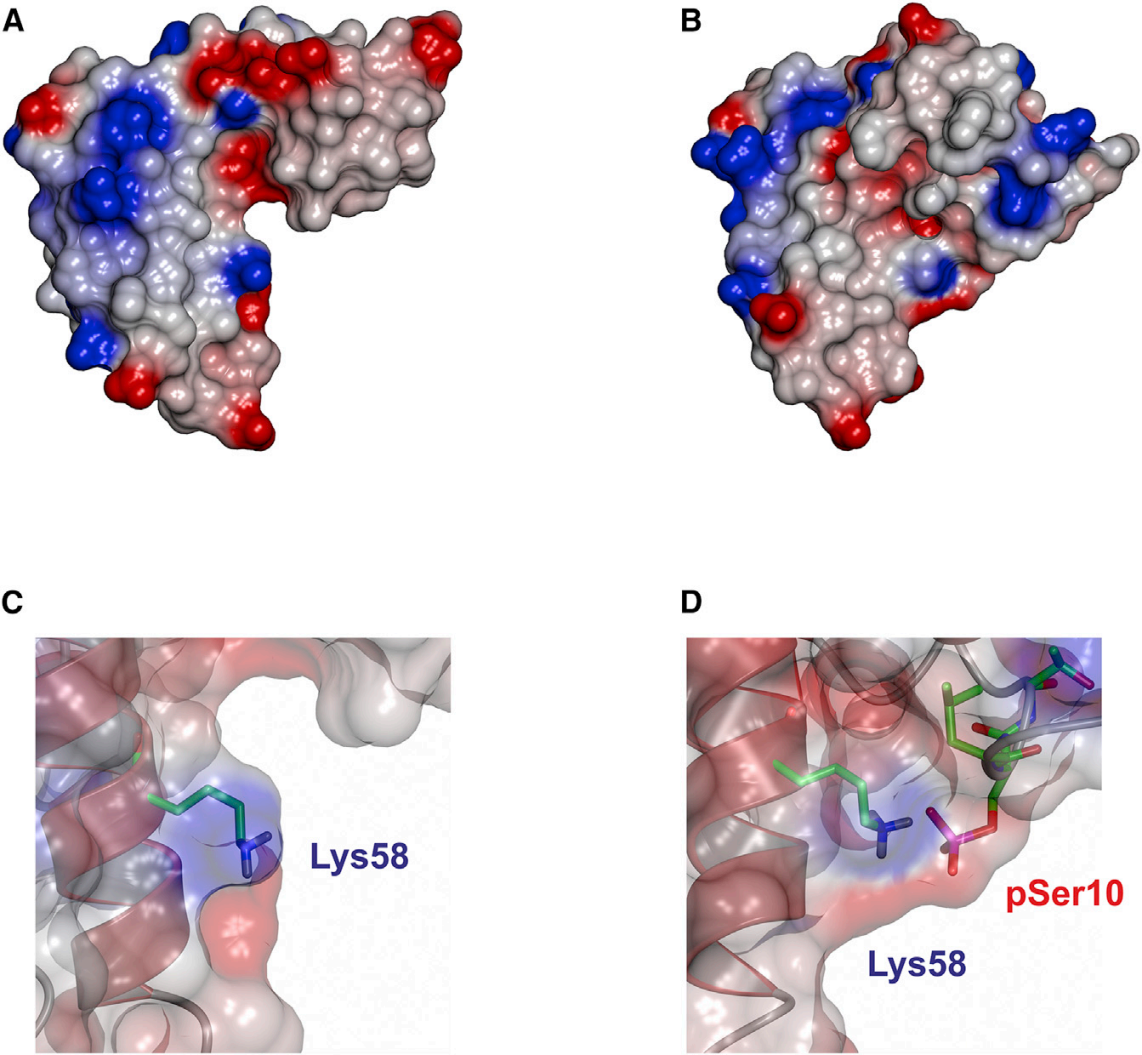
Phosphorylation of Ser10 Triggers a Conformational Switch (A and B) Surface representation of the lowest-energy conformers of CSP_1-100_ and pCSP_1-100_. (C) Close-up view of Lys58, represented as sticks, showing the surface-exposed positively charged patch in CSP_1-100_. (D) Phosphorylation triggers the interaction of phospho-Ser10 and Lys58, altering the surface charge distribution in this region.

The α1 helix contains the Ser10 residue, which is phosphorylated in vivo and which modulates CSP's cellular functions (Collins et al., 2005, Evans and Morgan, 2005, Evans et al., 2001). To gain insight into how phosphorylation affects CSP structure, purified ^13^C/^15^N CSP_1-100_ was incubated with MgATP and protein kinase A (PKA). Parallel incubation using unlabeled proteins showed that under these conditions, rapid and efficient phosphorylation on only Ser10 was achieved, as determined by γ^32^-ATP incorporation and mass spectrometry (Figure S4). The incubation mixture containing ^13^C/^15^N CSP_1-100_, MgATP, and PKA was used without further purification for structure determination. Triple-resonance heteronuclear NMR spectroscopy with non-uniform sampling (NUS) was then performed, allowing full data collection for spectral assignment in a short space of time. The spectra revealed significant changes to the chemical shifts for various residues, notably those around Ser10 and Ser81 (Figures 1B and 1C). Based on the mass spectrometry data, the chemical shift effects around S10 are a direct result of Ser10 phosphorylation, whereas those around Ser81 indicate a possible structural change in the loop connecting helices α5 and α6. Backbone resonance assignments for all 100 assignable amino acid residues were obtained, and the structure of pCSP_1-100_ was calculated using 3,301 distance and dihedral angle restraints. Strikingly, the structure of serine10-phosphorylated CSP_1-100_ reveals an order-to-disorder transition in the conformation of helix α1, which in turn triggers the interaction of the newly disordered N terminus with the J-domain helix α4 (Figures 2C, 2D, and S2B). This conformational phospho-switch results in a more compact overall structure of pCSP_1-100_ with significantly altered surface charge distribution (Figures 3A and 3B). Notably, the ionic interaction between the negatively charged phosphate group on phospho-Ser10 and the positively charged ɛ-amino group of Lys58 stabilizes and sequesters the N-terminal region of CSP (Figures 3C and 3D), which also brings the N-terminal region into much closer proximity to Ser81, hence, explaining the significant chemical shift changes in this region. The interaction between phospho-Ser10 and Lys58 is corroborated by the observation of a network of nuclear Overhauser effects (NOEs) involving the surrounding residues, including phospho-Ser10 to Ser81/Leu82, and Val19 to Glu59/Ile60/Ala63. Unambiguous direct NOEs between phospho-Ser10 and Lys58 are not observed, as the distances between the non-exchangeable protons in the two residues are over 5 Å and, hence, expected to give rise to very weak NOEs. The relatively small chemical shift change in the ^15^N-HSQC spectrum around residue Lys58 compared with Ser81 is explained by the lack of conformational change in helix α4.

## Discussion

**Figure 4:**
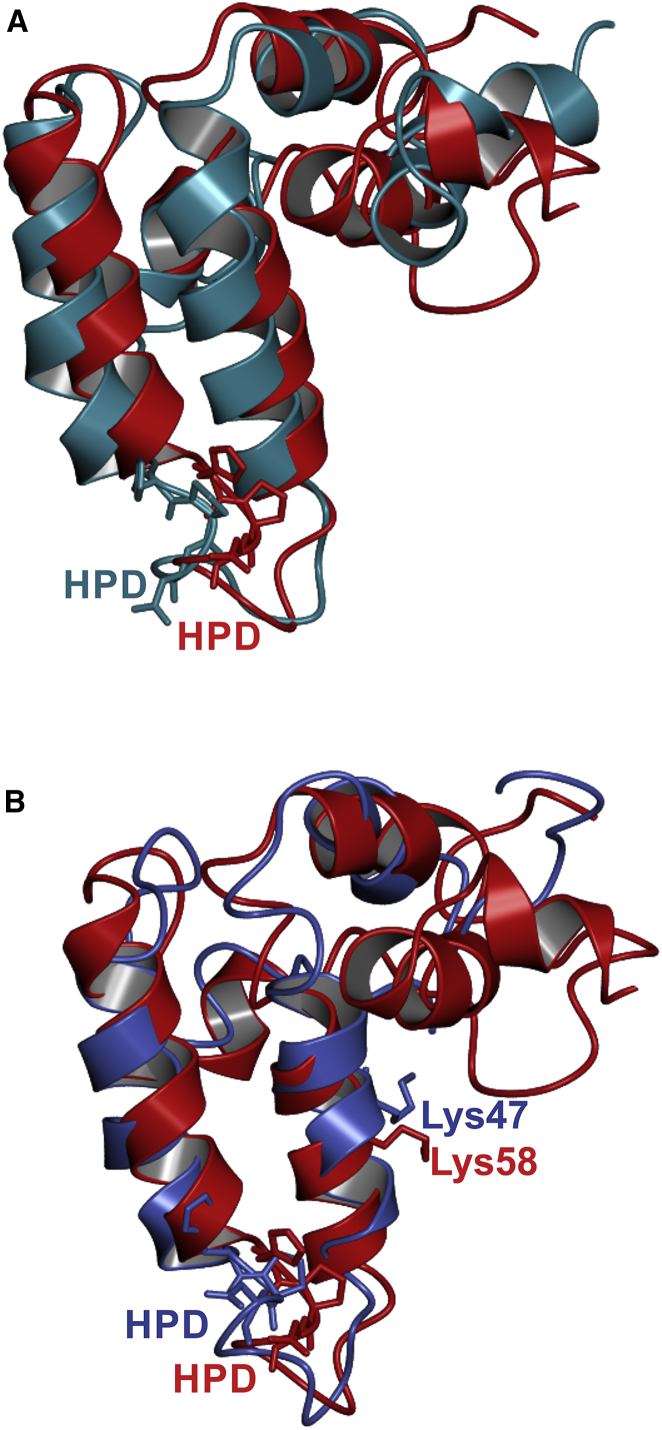
Implications of CSP Phosphorylation for Interactions with Hsc70 and Ubiquitin Ligases (A) Overlay of CSP_1-100_ (blue) and pCSP_1-100_ (red), with the conserved HPD motif represented as sticks. (B) Overlay of *E. coli* DnaJ (PDB: 1BQ0; blue) with human pCSP_1-100_ (red), with the conserved Lys58 and Lys48 residues, respectively, and HPD motifs represented as sticks.

The conformational phospho-switch reported here provides a structural basis for the previously established effects of Ser10 phosphorylation on CSP function. By destabilizing the N-terminal α1 helix and reducing its accessibility, phosphorylation would weaken protein-protein interactions involving this region, potentially explaining how Ser10 phosphorylation reduces CSP binding to syntaxin and synaptotagmin (Evans and Morgan, 2002, Evans et al., 2001). In contrast, the structure of the J domain and the accessibility of the HPD motif required for Hsp70 activation are unaffected by Ser10 phosphorylation (Figure 4A), thus revealing why CSP phosphorylation has no effect on Hsp70 interactions (Evans et al., 2001). Finally, the new interface created jointly by the phosphorylated N terminus and α4 helix (Figure 3B) provides a novel scaffold for protein and/or lipid interactions that could explain the effects of Ser10 phosphorylation on fusion pore expansion during exocytosis (Chiang et al., 2014, Evans and Morgan, 2002, Evans et al., 2001, Prescott et al., 2008).

Phosphorylation-induced order/disorder transitions, as shown here for CSP, are becoming increasingly recognized as regulatory switches that control protein function. For example, phosphorylation of retinoblastoma protein on Ser608 causes the disordered loop containing this residue to interact with the binding pocket for the E2F transactivation domain, thus inhibiting E2F binding (Burke et al., 2012). In addition, multi-site phosphorylation of folded pentameric nucleophosmin has been shown to cause electrostatic repulsion between the protomers and a transition to unfolded monomers, thereby destabilizing binding sites that exist in the oligomeric protein (Mitrea et al., 2014). Finally, a phosphorylation-induced disorder-to-order transition in 4E-BP2 has recently been shown to reduce eIF4E binding by sequestering a helical binding motif into a β strand (Bah et al., 2015).

The N-terminal domain of CSP is phosphorylated in vivo in humans, rodents, flies, and worms (Collins et al., 2005, Evans and Morgan, 2005, Evans et al., 2001, Hilger et al., 2009, Zielinska et al., 2009), indicating that phospho-regulation of CSP is as evolutionarily conserved as its role in preventing neurodegeneration. Given that 36 of the 41 DnaJ proteins encoded by the human genome are serine/threonine phosphorylated (Hornbeck et al., 2015), the CSP phospho-switch revealed here could be a general mechanism for conformational regulation of DnaJ/Hsp40 chaperones. Indeed, the Lys58 residue that interacts with phospho-Ser10 in CSP has long been recognized to be among the most highly conserved residues in DnaJ proteins (Hennessy et al., 2005) (Figure 4B), although the reason for this conservation has been unclear. Furthermore, Lys58 in CSP is a ubiquitination site (Wagner et al., 2011), as are the orthologous Lys residues in human DNAJA1 and DNAJB1. The close interaction of phospho-Ser10 with Lys58 revealed here would likely impede access by E3 ligases, thereby antagonizing CSP ubiquitination. Given that phosphorylation of 4E-BP2 has recently been shown to inhibit Lys57 ubiquitination by triggering a disorder-to-order transition (Bah et al., 2015), the phospho-switch reported here may represent an alternative mechanism for regulating protein conformation by reciprocally antagonistic posttranslational modifications.

## Experimental Procedures

### Expression and Purification of CSP

Full-length CSP_1-198_ in the pQE30 vector (QIAGEN) has been previously described (Evans et al., 2001) and was used to prepare the CSP14CS, CSP_137-198_, and CSP_1-112_ constructs via site-directed mutagenesis. CSP_1-100_ was synthesized (Geneart; Life Technologies) based on the human coding sequence and codon optimized for expression in *Escherichia coli* and subcloned into the pE-Sumopro Kan expression vector (LifeSensors). Expression of recombinant CSP was induced in *E. coli* BL21 Star (Invitrogen) competent cells using 1 mM isopropyl β−D-1-thiogalactopyranoside at 18°C for 18 hr. Uniformly isotope-labeled CSP was expressed in M9 minimal media with ^15^NH_4_Cl and/or ^13^C_6_-glucose as the sole nitrogen and carbon sources, respectively. Cells were harvested by centrifugation and resuspended in lysis buffer containing 20 mM Tris (pH 7.5), 500 mM NaCl, 20 mM imidazole with protease inhibitors (complete mini EDTA-free protease inhibitor cocktail tablets; Roche). After lysis by cell disruption, the soluble fraction was isolated by centrifugation at 27,000 × *g* for 45 min. The supernatant was applied to a charged HisTrap FF 5 ml affinity column (GE Healthcare), washed with 20 mM Tris (pH 7.5), 500 mM NaCl, 50 mM imidazole, and purified protein eluted with a linear imidazole gradient from 50 mM to 500 mM. The His-SUMO tag on CSP_1-100_ was removed by incubation with recombinant ULP-1 overnight at 4°C. The CSP_1-100_ protein was subjected to gel filtration through a Superdex-75 column (GE Healthcare) equilibrated with 20 mM 2-(N-morpholino)ethanesulfonic acid (pH 6.5), 150 mM NaCl.

### In Vitro Phosphorylation

Purified CSP_1-100_ was phosphorylated by mixing in a 340:1 molar ratio with protein kinase A catalytic subunit (Sigma-Aldrich), 1 mM DTT, 10 mM MgCl_2_, 0.5 mM EDTA, and 1 mM ATP. For analysis of phosphorylation kinetics, mixtures were supplemented with 3 μCi of radiolabeled γ^32^-ATP per 50 μl reaction and incubated for various times before stopping the reaction by addition of boiling 2× Laemmli buffer (Sigma-Aldrich). Samples were run on pre-cast Novex SDS-PAGE gels (Invitrogen), stained with Coomassie blue, dried, and exposed to phosphor screens overnight before imaging on a Phosphorimager Si (GE Healthcare). For NMR spectroscopy and mass spectrometry analyses, in vitro phosphorylation mixtures were prepared using non-radiolabeled ATP and incubated for 4 hr to ensure the reaction was complete.

### Estimation of Native Molecular Mass

Analytical ultracentrifugation was performed at the Astbury Center for Structural Molecular Biology, University of Leeds. CSP protein samples were spun at 50,000 rpm at 20.1°C for 9 hr for sedimentation velocity analysis, during which 98 absorbance scans at 279 nm were performed and used to estimate the native molecular mass. Size-exclusion chromatography-multiple-angle laser light scattering analysis was performed using a Dawn Heleos instrument at a laser wavelength of 658 nm.

### Mass Spectrometry

Phosphorylation site mapping was performed at the FingerPrints' Proteomics Facility, University of Dundee. PKA-phosphorylated CSP_1-100_ protein was separated by SDS-PAGE, digested with trypsin, and then extracted before being applied to an nLC liquid chromatography system (Dionex/LC Packings) coupled to a 4000 QTRAP mass spectrometer (Applied Biosystems/Sciex). Mass spectrometry data were filtered by removing missed cleavages and employing a 1% false discovery rate.

### NMR Spectroscopy

All spectra were acquired at 298 K on Bruker Avance III 600 MHz and 800 MHz spectrometers. For non-phosphorylated CSP_1-100_, sequence-specific backbone resonance assignment was obtained using standard multidimensional heteronuclear NMR experiments: HNCA, HN(CO)CA, HNCACB, CBCA(CO)NH, HNCO, HNCACO, HBHANH, HBHA(CO)NH. Side-chain assignments were obtained from a 3D HCCH total correlation spectroscopy (HCCH-TOCSY) experiment. NOEs were derived from 3D ^15^N- and ^13^C-edited NOE spectroscopy (NOESY)-HSQC experiments with 130 ms mixing time. For pCSP_1-100_, sequence-specific backbone resonance assignment was obtained using the multidimensional heteronuclear NMR experiments as described above, with NUS. Side-chain assignments were obtained from a 3D HCCH-TOCSY experiment. NOEs were derived from 3D ^15^N- and ^13^C-edited NOESY-HSQC experiments with 140 ms mixing time.

### NMR Assignments and Structure Calculations

All NMR spectra were processed with TopSpin (Bruker) and analyzed using the CCPN Analysis package (Vranken et al., 2005). Backbone torsion angles were derived from analysis of Hα, Cα, Cβ, and C′ chemical shifts using the DANGLE program (Cheung et al., 2010). All structure calculations were carried out using the Aria package (Rieping et al., 2007) with the IUPAC PARALLHDGv5.3 and TOPALLHDGv5.3 parameter sets. Structural statistics are summarized in Table 1.

## Author Contributions

P.P., G.R.P., and A.M. performed protein purification and biochemical experiments; P.P. and L.Y.L. performed NMR experiments; P.P. performed structure calculations; P.P., L.Y.L., R.D.B., and A.M. analyzed and interpreted the data. A.M., L.Y.L., and R.D.B. conceived and designed the study. P.P. and A.M. wrote the manuscript with input from all authors.

## Figures and Tables

**Table 1:** NMR and Refinement Statistics for Protein Structures

	CSP 1-100	pCSP 1-100
**NMR Distance and Dihedral Constraints**		

Distance constraints		
Total NOE	2,450	3,120
Intra-residue	851	968
Inter-residue	1,599	2,152
Sequential (|*i* – *j*| = 1)	655	839
Medium-range (2 ≤ |*i* – *j*| ≤ 4)	530	758
Long-range (|*i* – *j*| ≥ 5)	414	555
Total dihedral angle restraints	187	181
ϕ	92	90
ψ	95	91

**Structure Statistics**		

Violations (mean and SD)		
Distance constraints (Å)	0.08 ± 0.06	0.07 ± 0.07
Dihedral angle constraints (°)	1.15 ± 0.88	1.44 ± 1.10
Max. dihedral angle violation (°)	4.79	5.57
Max. distance constraint violation (Å)	0.72	1.13
Deviations from idealized geometry		
Bond lengths (Å)	0.0041 ± 0.00013	0.0050 ± 0.0001
Bond angles (°)	0.59 ± 0.012	0.65 ± 0.016
Impropers (°)	1.54 ± 0.056	1.59 ± 0.067
Average pairwise root-mean-square deviation^a^ (Å)		
Heavy	0.47	0.49
Backbone	0.14	0.20
Ramachandran statistics^b^		
Most favored/additionally allowed/generously allowed (%)	88.2/11.8/0.0	80.6/18.3/1.1

a Statistics are calculated and averaged over an ensemble of the 20 lowest-energy water-refined structures out of 100 calculated structures.

b Ramachandran statistics calculated using PROCHECK.

## Accession Numbers

Coordinates and chemical shifts have been deposited in the PDB and Biological Magnetic Resonance Bank under accession codes PDB: 2N05 and BMRB: 25515 for CSP_1-100_, and PDB: 2N04 and BMRB: 25514 for pCSP_1-100_.

## Supplemental Information

Document S1. Figures S1–S4

Document S2. Article plus Supplemental Information
