# Supplementary material for: Phosphorylation of Cysteine String Protein Triggers a Major Conformational Switch
Source: Structure. 2016 Aug 2;24(8):1380–6. doi: 10.1016/j.str.2016.06.009 (PMC4975591; doi:10.1016/j.str.2016.06.009)
Supplement: Document S1. Figures S1–S4 [file mmc1.pdf]

Structure, Volume *24*

Supplemental Information

Phosphorylation of Cysteine String Protein Triggers a Major Conformational Switch

Pryank Patel, Gerald R. Prescott, Robert D. Burgoyne, Lu-Yun Lian, and Alan Morgan

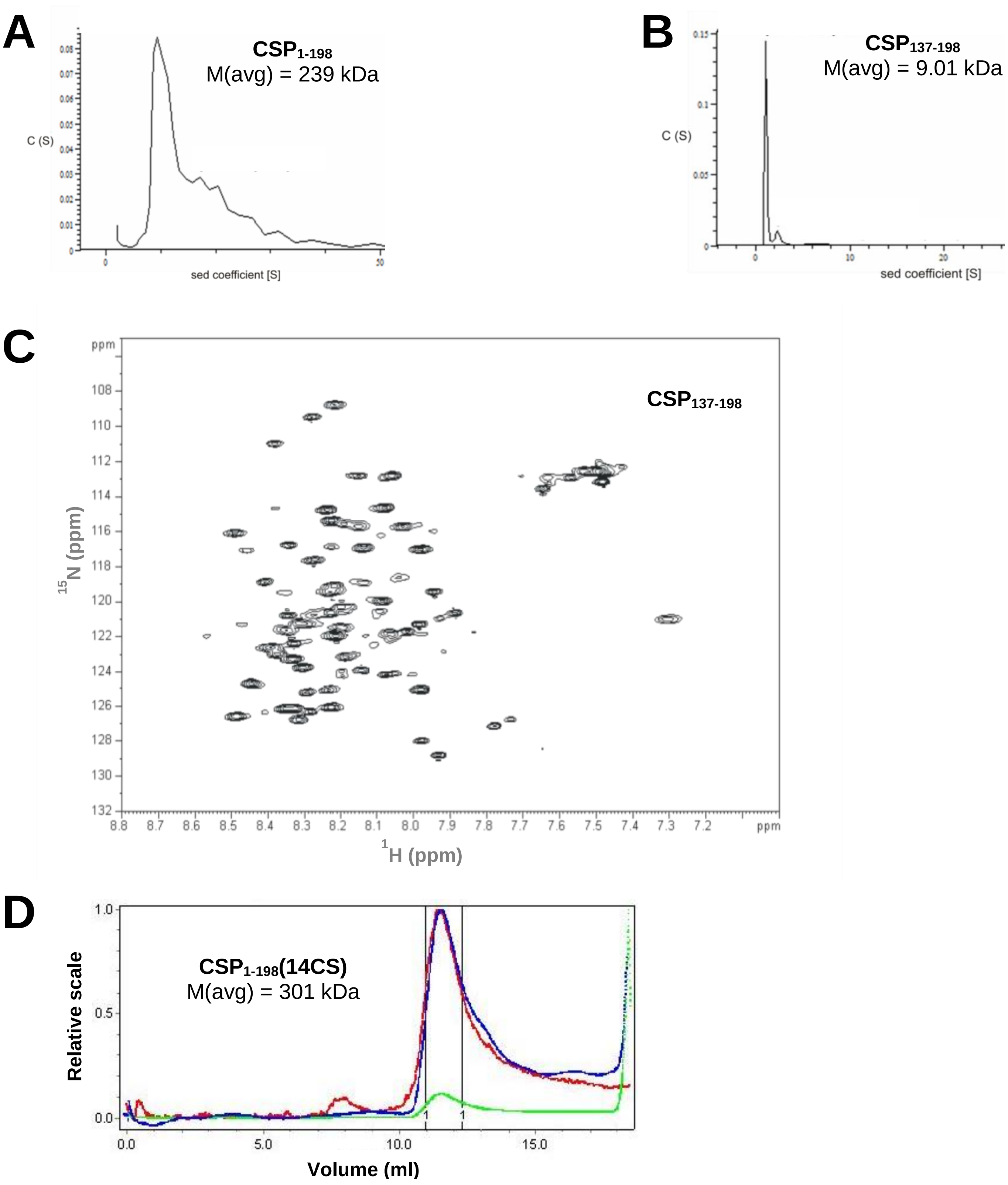

Fig. S1, related to Fig. 1: Characterisation of recombinant CSP constructs
a) Analytical ultracentrifugation of full length CSP$_{1-198}$. b) Analytical ultracentrifugation of CSP$_{137-198}$. c) ^{1}H-^{15}N HSQC spectra of CSP$_{137-198}$. d) Size exclusion chromatography - multiple angle laser light scattering analysis of full length CSP$_{1-198}$ with all 14 cysteines mutated to serines (14CS).

A

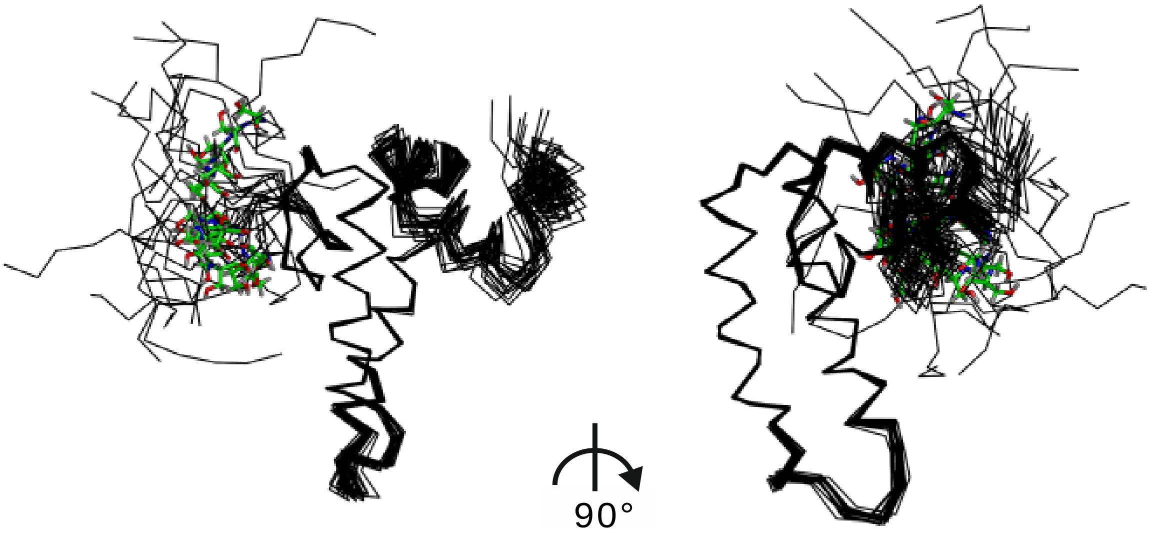

B

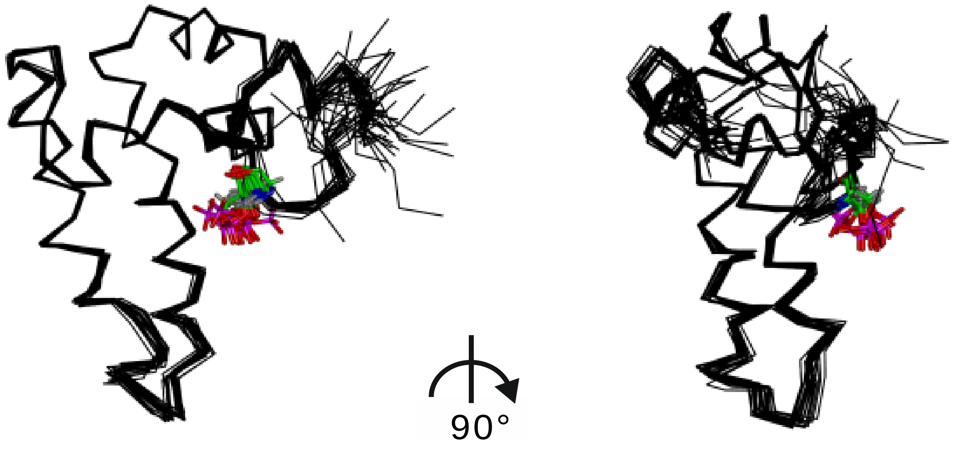

Fig. S2, related to Fig. 2: Structures of unphosphorylated and Ser10-phosphorylated $CSP_{1\text{-}100}$.

Ensembles of the 20 lowest energy conformers of $CSP_{1\text{-}100}$ (a) and $pCSP_{1\text{-}100}$ (b) displayed as Cα backbone representations; two different views differing by 90° are shown for each, with Ser10 represented as sticks.

A

B

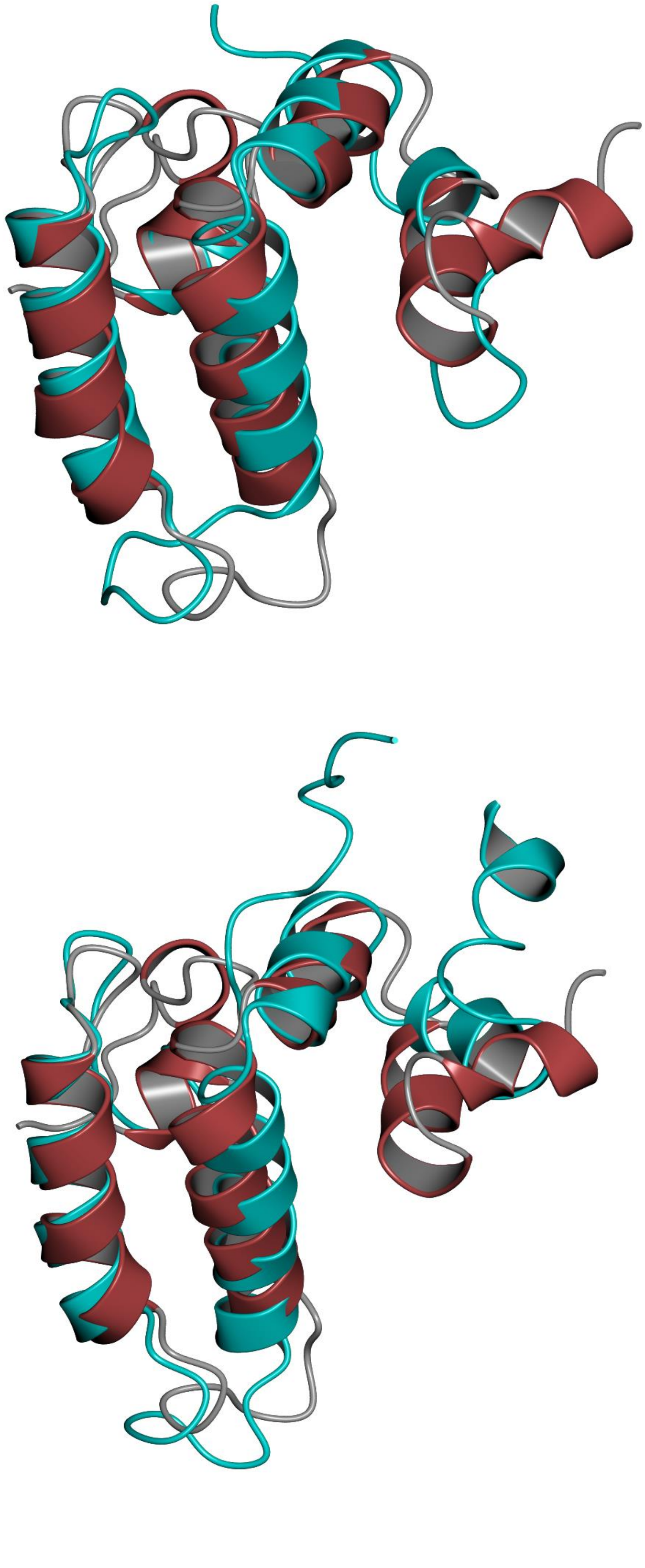

Fig. S3, related to Fig. 2: Structural similarities between CSP$_{1-100}$ and DnaJ/Hsp40 proteins in the unphosphorylated state.

a) Overlay of the lowest energy conformer of CSP$_{1-100}$ (red) with the J domain of yeast Sis1 protein (PDB code: 4RWU, cyan). b) Overlay of the lowest energy conformer of CSP$_{1-100}$ (red) with CSP$_{5-100}$ (PDB code: 2CTW, blue).

A

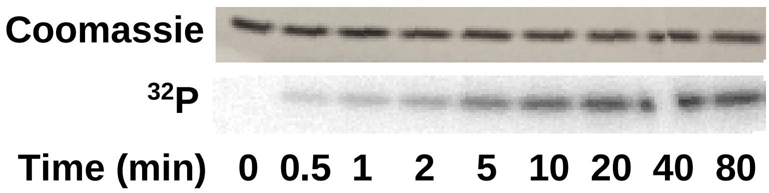

B

Sequence	# PSMs	Modifications	IonScore	Exp Value	Charge	MH+ [Da]	ΔM [ppm]	RT [min]	# Missed Cleavages
SLsTSGESLYHVLGLDK	153	S10(Phospho)	63	0.000	2	1885.895	0.31	67.11	0
sLsTSGESLYHVLGLDK	33	N-Term(Acetyl); S10(Phospho)	50	0.000	2	1927.906	0.52	77.20	0
sLsTSGESLYHVLGLDK	33	S8(Phospho); S10(Phospho)	40	0.002	2	1965.861	0.16	76.41	0

Fig. S4, related to Fig. 3: *in vitro* phosphorylation of CSP_{1-100}.
a) Kinetics of γ^{32}-ATP incorporation following incubation of CSP_{1-100} with protein kinase A and radiolabelled MgATP. Samples were run on SDS-PAGE and visualised by Coomassie Blue staining (*Coomassie*, upper panel) and phosphorimaging (*^{32}P*, lower panel). Images shown are cropped to remove unnecessary blank space. b) Mass spectrometric identification of phosphorylation sites on CSP_{1-100} after incubation with protein kinase A and MgATP.
